# Symptom clusters mediate anxiety/depression effects on quality of life in neuromyelitis optica spectrum disorders: a cross-sectional mediation analysis

**DOI:** 10.3389/fneur.2026.1753642

**Published:** 2026-06-18

**Authors:** Qing Yang, Lin Wei, Lixin Wang, Hao Liang, Haoyou Xu, Xiaopei Zhang, Zhuyun Liu

**Affiliations:** Guangdong Provincial Hospital of Chinese Medicine, Guangzhou University of Chinese Medicine, Guangzhou, China

**Keywords:** anxiety, depression, neuromyelitis optica spectrum disorder, NMOSD, QOL, quality of life, symptom clusters

## Abstract

**Aims and objectives:**

To investigate the mediating effects of symptom clusters on the relationship between anxiety/depression and quality of life (QOL) among Chinese patients with neuromyelitis optica spectrum disorder (NMOSD).

**Background:**

NMOSD is a rare and severe inflammatory demyelinating disease of the central nervous system, predominantly affecting the optic nerves and spinal cord. Patients with NMOSD often experience significant neurological disability and a high burden of psychiatric comorbidities, including anxiety and depression, which are associated with lower QOL. Although anxiety and depression are established correlates of QOL in NMOSD, the pathways linking them through symptom clusters remain unexplored.

**Design:**

A cross-sectional design.

**Methods:**

A total of 140 participants diagnosed with NMOSD were recruited through online convenience sampling. Data were collected using standardized tools, including the Patient Health Questionnaire-9 (PHQ-9) and Generalized Anxiety Disorder-7 (GAD-7) for assessing anxiety and depression, the 36-item Short-Form Health Survey (SF-36) for evaluating QOL, and a self-administered NMOSD symptom scale for identifying symptom clusters. Mediation analyses were performed using the SPSS PROCESS macro with bias-corrected bootstrap methods.

**Results:**

Patients with NMOSD had a QOL score of 97.76 ± 8.24. Educational level and primary caregivers were identified as factors associated with QOL. This study revealed significant negative correlations between anxiety/ depression and QOL, with mediation observed through distinct symptom clusters. Anxiety exhibited complete mediation via somatosensory and motor symptom clusters, with partial mediation through bladder-rectal symptoms. Depression demonstrated complete mediation through somatosensory, motor, visual-memory, and bladder-rectal symptom clusters. Notably, sleep-related symptom clusters did not show significant mediating effects.

**Conclusion:**

Symptom clusters may mediate the associations between anxiety/depression and QOL. The study underscores the importance of considering both psychological distress and physical symptoms in relation to QOL in NMOSD patients. Future research is needed to validate these findings in larger, diverse cohorts and to explore targeted interventions aimed at optimizing functional outcomes in this population.

## 1 Background

Neuromyelitis optica spectrum disorder (NMOSD) is a rare inflammatory demyelinating disease of the central nervous system predominantly affecting the optic nerves and spinal cord. Its pathogenesis is closely associated with aquaporin-4 (AQP4) antibodies, which target astrocytes and drive autoimmune injury (1, 2). Globally, NMOSD has a prevalence of approximately 1.51 per 100,000, with higher rates in non-Caucasian populations and a marked female predominance (3, 4). In China, the annual incidence is 0.374 per 100,000, indicating a substantial disease burden (5, 6). NMOSD is characterized by a relapsing course (80–90%), often leading to cumulative and irreversible neurological deficits such as visual loss, motor dysfunction, and bladder/bowel disturbances (1, 7). As a chronic disabling condition, NMOSD imposes a substantial burden on quality of life (QOL) (8–10).

Psychological comorbidities are common in NMOSD, with approximately 46.1% experiencing depression and 45% reporting anxiety (11, 12). The severity of these mood disorders correlates significantly with the degree of neurological disability. Existing evidence identified anxiety and depression as key predictors of QOL deterioration in NMOSD, associated with impaired interpersonal relationships, sexual dysfunction, suicidal ideation, and reduced treatment adherence (9, 13–15). Consequently, proactive management of psychological symptoms is critical to preserving functional independence in daily living. However, these symptoms remain under-recognized and undertreated, particularly in Chinese populations (16). According to the Theory of Unpleasant Symptoms, psychological distress may exacerbate the perception and severity of physical symptoms, thereby worsening QOL (17). However, empirical evidence on pathways in NMOSD remains limited. Investigations are thus imperative to delineate the underlying pathways through which psychological exacerbate somatic symptom burden and compromise QOL, thereby informing precision management strategies.

Recent advancements in symptom cluster research have provided novel insights into understanding complex symptom interactions in chronic diseases (18). Symptom clusters refer to symptom constellations characterized by co-occurrence, shared underlying pathophysiology, and synergistic effects on patient outcomes (19). The identification of symptom clusters serves as a critical dimensionality reduction approach in real-world clinical practice, enabling simplification of intricate symptom networks. In NMOSD, delineating symptom clusters facilitates a deeper understanding of inter-symptom relationships, thereby informing evidence-based symptom management strategies. Our prior research employing network analysis methodology identified six distinct symptom clusters in NMOSD patients (20): somatosensory symptom clusters, motor symptom clusters, visual-memory symptom clusters, bladder-rectal symptom clusters, sleep symptom clusters, and neuropsychiatric symptom clusters. However, current research paradigms predominantly focus on isolated symptom-outcome associations, failing to elucidate the systemic impacts of symptom clusters (21–23).

Although anxiety and depression are established predictors of QOL, their underlying mechanisms remain unclear (9). Previous studies have primarily examined direct effects, with limited attention to indirect pathways through symptom burden (24), whereas depression could restrict social participation through fatigue and cognitive impairment, thereby establishing a self-perpetuating cycle of symptom-psychopathology interactions (25). However, no prior studies have systematically investigated the mediating effects of symptom clusters in the anxiety/depression-QOL pathway. To address this gap, this study constructs a mediation model with anxiety/depression as independent variables and five non-neuropsychiatric symptom clusters (somatosensory, motor, visual-memory, bladder-rectal, and sleep-related symptoms) as mediators. We aim to resolve two pivotal questions:

- Do non-neuropsychiatric symptom clusters mediate the relationship between anxiety/depression and QOL?
- Which specific symptom clusters demonstrate significant mediating effects?

The innovation of this study lies in being the first to conceptualize non-neuropsychiatric symptom clusters as composite mediators, transcending conventional single-symptom approaches. This paradigm shift provides novel evidence for psychophysiological interaction mechanisms in NMOSD and establishes a theoretical foundation for developing integrated interventions centered on symptom cluster management.

## 2 Methods

### 2.1 Design, participants, and procedure

This study employed a cross-sectional design and was reported in accordance with the Strengthening the Reporting of Observational Studies in Epidemiology (STROBE) guidelines (26). It was also registered in the Chinese Clinical Registry Center on November 11, 2023 (No. ChiCTR2300077533). We conducted an online survey in China between October 10 to 30, 2023. Participants were recruited online using the convenience sampling method. Inclusion criteria were as follows: (1) diagnosed with NMOSD by neurologists based on European Federation of Neurological Societies guidelines on diagnosis and management of neuromyelitis optica (27); (2) aged 18 years and over; and (3) informed consent. Participants were excluded if they were: (1) pregnant or breastfeeding women; (2) combined with other serious, chronic comorbidities or acute illnesses, such as malignant tumors, severe liver/renal dysfunctions, or cardiovascular disease; (3) diagnosed with mental illness or dementia and unable to communicate verbally.

The survey was created and distributed using Wenjuanxing software. It included two sections: instructions and socio-demographic/clinical data. Hosted on the WeChat platform,^1^ the questionnaire was accessible to 206 NMOSD patients from the Second Affiliated Hospital of Guangzhou University of Chinese Medicine. Participants could complete it anonymously via computer, tablet, or phone by clicking a link or scanning a QR code. Family members assisted those unable to complete it themselves. To prevent duplicates, one submission per device was allowed. Incomplete responses were prompted before submission, and only fully completed questionnaires were accepted. The questionnaire was sent twice to the WeChat group, with a one-week SMS reminder for non-respondents. A total of 151 responses were collected, yielding 140 valid ones (92.72% effective rate). The participant selection process is detailed in Supplementary File S1.

### 2.2 Sample size

Sample size was calculated using GPower 3.1 (28). Presuming a medium effect size of *f^2^* = 0.15, a statistical power of 1 − *β* = 0.8, and a significance level of *α* = 0.05, with seven predictor variables, requiring 103 participants (29). Accounting for 10% invalid responses, 114 participants were targeted. Final enrollment included 140 valid responses.

### 2.3 Measures

#### 2.3.1 Covariables: social-demographic characteristics

The social-demographic variables included age, gender, educational level, employment status, marital status, primary caregiver, insurance type, and personal monthly income. Health-related behavior variables included smoking status, drinking status and activities of daily living (ADL). Disease-related variables included duration of NMOSD, number of relapses, and polypharmacy.

The Barthel Index was utilized to evaluate activities of daily living (ADL) performance in this research (30). It consists of 10 elements: eating, washing, grooming, dressing, bowel control, bladder control, toileting, bed-to-chair transfer, walking, and stair climbing. The maximum score is 100, where higher scores reflect greater autonomy in daily activities. Prior studies have confirmed the ADL scale’s high reliability and validity among Chinese people (31).

#### 2.3.2 Independent variable: anxiety and depression

The Patient Health Questionnaire-9 (PHQ-9) (32) and Generalized Anxiety Disorder-7 (GAD-7) (33) were used to evaluate depression and anxiety. These tools are based on the Diagnostic and Statistical Manual of Mental Disorders, Fourth Edition (DSM-IV) criteria and assess symptoms experienced over the past 2 weeks. Each item is scored on a 4-point scale from 0 (not at all) to 3 (nearly every day). Both scales have been validated in China and show good reliability and validity (34).

#### 2.3.3 Dependent variable: QOL

The 36-item Short-Form Health Survey (SF-36) was utilized to assess participants’ QOL (35). This instrument evaluates eight dimensions of QOL, including physical functioning (10 items), role limitations due to physical health (4 items), bodily pain (2 items), general health perceptions (5 items), vitality (4 items), social functioning (2 items), role limitations due to emotional issues (3 items), and mental health (5 items). Additionally, it includes one question regarding changes in health status over the past year. Item scores are normalized on a scale from 0 (indicating poor health) to 100 (indicating optimal health). The total possible score for the scale is 145, with higher scores reflecting better QOL. The scale has been widely used in China and has demonstrated good reliability and validity (35).

#### 2.3.4 Mediator: symptom clusters

A customized NMOSD symptom scale was used to assess patients’ symptom experiences over the past week. The scale was developed based on the Revised Symptom Management Conceptual Model (36), a widely used theoretical framework for symptom assessment and management.

The development of the scale followed a rigorous multi-step process. First, an initial item pool was generated through comprehensive literature review and qualitative group discussions. Second, a two-round Delphi expert consultation was conducted to ensure content relevance, clarity, and comprehensiveness. Third, a pilot survey was performed to further refine item wording and structure. Through this iterative process, a final 29-item NMOSD symptom scale was established. The NMOSD symptom scale has been used in our prior published work based on the same patient population framework (20), and the present study represents a further application focusing on symptom cluster–based mediation pathways.

Each item is assessed using a dichotomous response (“yes”/“no”). For symptoms endorsed as “yes,” frequency, severity, and distress are further evaluated. Frequency and severity are measured using 4-point Likert scales (frequency: 1 = “rarely” to 4 = “always”; severity: 1 = “mild” to 4 = “very severe”), while distress is rated on a 5-point Likert scale (0 = “not at all” to 4 = “very much”). In this study, the scale demonstrated excellent internal consistency (Cronbach’s *α* = 0.976) and good content validity (content validity index = 0.96; item-level CVI = 0.85–1.00).

### 2.4 Ethical considerations

The study was approved by the Ethics Committee of the Second Affiliated Hospital of Guangzhou University of Chinese Medicine (approval No. BE2023-096-01). Additionally, informed consent was obtained from all participants.

### 2.5 Data analysis

Data analysis was performed using SPSS 26.0 (IBM Corp., Armonk, NY, USA). The online questionnaire was designed to ensure submission only after complete completion, eliminating missing values in the dataset. Variables were considered normally distributed if skewness≤2 and kurtosis≤4 (37), which was confirmed for all variables. The distribution of QOL across sociodemographic characteristics was analyzed using independent-sample t-tests and one-way ANOVA. Mediating effects were assessed using the SPSS PROCESS macro with a bias-corrected (*BC*) bootstrap method (5,000 samples) (38). A significant indirect effect was indicated by a 95% BC bootstrap confidence interval (*CI*) that did not include zero (*p* < 0.05). Complete mediation was identified if the independent variable was no longer associated with the dependent variable when controlling for the mediator; otherwise, partial mediation was considered. Covariates were selected based on the results of univariate analyses; only variables showing significant associations with QOL were included in the mediation models to ensure model parsimony and avoid over-fitting. All statistical tests were two-tailed, with significance set at *p* < 0.05.

## 3 Results

### 3.1 Social-demographic characteristics

The study comprised 140 participants with a mean age of 42.20 years (*SD* = 12.46) and an average relapse frequency of 3.13 episodes (*SD* = 3.56). The mean disease duration was 60.59 months (*SD* = 55.74), with 52.9% of participants prescribed combination therapy involving five or more medications. The sample was predominantly female (87.9%) and married (74.3%). Notably, 58.57% reported independent living status (either self-care or no caregiver support). Health-related behaviors revealed a low prevalence of tobacco (93.6% never smokers) and alcohol consumption (92.9% never drinkers). In addition, 55.7% earn monthly incomes below 3,000 RMB, with 92.1% receiving medical insurance reimbursement for treatment costs (see Table 1).

**Table 1:** Social-demographic characteristics (*n* = 140).

Variables	*N* (%)/M ± SD	QOL		
		M ± SD	*t/F*	*p*
Age (years)	42.20 ± 12.46			
Sex			−1.837^a^	0.86
Male	17 (12.1)	94.35 ± 8.03		
Female	123 (87.9)	98.23 ± 8.19		
Educational level			4.619^b^	0.004
Primary school or below	7 (5.0)	95.85 ± 9.19		
Secondary school	30 (21.4)	100.87 ± 7.40		
High school	29 (20.7)	93.48 ± 8.42		
University or above	74 (52.9)	98.36 ± 7.83		
Employment			1.087^b^	0.34
Mental workers	67 (47.9)	96.79 ± 7.79		
Manual workers	48 (34.3)	98.23 ± 9.21		
None	25 (17.9)	99.48 ± 7.34		
Marital status			1.116^b^	0.345
Unmarried	30 (21.4)	99.13 ± 8.72		
Married	104 (74.3)	97.11 ± 8.09		
Divorced	4 (2.9)	102.25 ± 7.59		
Widow	2 (1.4)	102.50 ± 9.19		
Primary caregivers			3.565^b^	0.031
Relatives	56 (40.0)	97.21 ± 8.34		
nanny	2 (1.4)	83.50 ± 4.95		
None/self-care	82 (58.6)	98.48 ± 7.95		
Insurance type			0.410^b^	0.801
Socialized medicine	5 (3.6)	100.60 ± 7.09		
UEBMI	86 (61.4)	97.47 ± 8.65		
URBMI	12 (8.6)	99.83 ± 9.81		
NRCMS	26 (18.6)	97.73 ± 7.98		
Self-pay	11 (7.9)	96.64 ± 3.29		
Personal monthly income (yuan)			0.794^b^	0.531
None	48 (34.3)	98.27 ± 7.77		
<3,000	30 (21.4)	96.37 ± 9.65		
3,000 ~ 5,999	39 (27.9)	98.33 ± 8.66		
6,000 ~ 9,999	13 (9.3)	95.46 ± 6.53		
>10,000	10 (7.1)	100.3 ± 5.95		
Smoking			0.614^b^	0.543
Never smoker	131 (93.6)	97.83 ± 8.32		
Former smoker	4 (2.9)	93.50 ± 8.96		
Current smoker	5 (3.6)	99.20 ± 5.01		
Drinking			0.023^b^	0.977
Never drinker	130 (92.9)	97.72 ± 8.35		
Former drinker	8 (5.7)	98.25 ± 7.98		
Current drinker	2 (1.4)	98.50 ± 0.71		
Polypharmacy (≥5)			0.892^a^	0.143
No	74 (52.9)	98.35 ± 7.58		
Yes	66 (47.1)	97.11 ± 8.94		
Number of relapses	3.13 ± 3.56			
Duration of disease (months)	60.59 ± 55.74			

UEBMI, urban employee-based basic medical insurance; URBMI, urban resident-based basic medical insurance; NRCMS, new rural cooperative medical scheme. ^a^Independent-sample *t* test. ^b^One-way ANOVA.

### 3.2 Scores of scales with dimensions

As shown in Table 2, the composite symptom clusters score demonstrated substantial variability (*M* = 53.52, *SD* = 42.43, range: 0–195), with somatosensory symptom clusters (*M* = 15.04, *SD* = 12.56, range: 0–48), motor symptom clusters (*M* = 9.41, *SD* = 11.61, range: 0–48), visual-memory symptom clusters (*M* = 9.11, *SD* = 9.07, range: 0–41), bladder-rectal symptom clusters (*M* = 9.23, *SD* = 13.16, range: 0–68), and sleep symptom clusters (*M* = 10.74, *SD* = 10.32, range: 0–36). Participants exhibited moderate psychological distress, with anxiety (*M* = 13.05, *SD* = 5.20, range: 7–28) and depression (*M* = 17.56, *SD* = 5.88, range: 9–36). Participants maintained preserved basic functioning in ADL (*M* = 28.77, *SD* = 3.00, range: 14–30), while demonstrating suboptimal QOL (SF-36: *M* = 97.76, *SD* = 8.24, range: 75–116).

**Table 2:** Scores of scales with dimensions.

Scales	*M*	*SD*	Range
Anxiety	13.05	5.20	7 ~ 28
Depression	17.56	5.88	9 ~ 36
QOL	97.76	8.24	75 ~ 116
Symptom clusters	53.52	42.43	0 ~ 195
Somatosensory symptom clusters	15.04	12.56	0 ~ 48
Motor symptom clusters	9.41	11.61	0 ~ 48
Visual-memory symptom clusters	9.11	9.07	0 ~ 41
Bladder-rectal symptom clusters	9.23	13.16	0 ~ 68
Sleep symptom clusters	10.74	10.32	0 ~ 36
ADL	28.77	3.00	14 ~ 30

### 3.3 Univariate analysis

According to the univariate analysis results presented in Table 1, significant differences emerged in educational level (*F* = 4.619, *p* = 0.004) and primary caregivers (*F* = 3.565, *p* = 0.031), no other variables (sex, employment, marital status, insurance type, personal monthly income, polypharmacy, health behaviors) showed significant differences (all *p* > 0.05).

### 3.4 Pearson correlation analysis

The analysis demonstrated statistically significant correlations among anxiety, depression, symptom clusters, and QOL. Specifically, anxiety (*r* = −0.222, *p* < 0.01), depression (*r* = −0.212, *p* < 0.05), somatosensory (*r* = −0.276, *p* < 0.01), motor (*r* = −0.296, *p* < 0.01), visual-memory (*r* = −0.239, *p* < 0.01), bladder-rectal (*r* = −0.265, *p* < 0.01), and sleep symptom clusters (*r* = −0.215, *p* < 0.05) all showed negative associations with SF-36 scores. Additionally, anxiety and depression exhibited positive correlations with all symptom cluster dimensions, ranging from *r* = 0.204 (bladder-rectal clusters, *p* < 0.05) to *r* = 0.443 (visual-memory clusters, *p* < 0.01) (see Table 3).

**Table 3:** Correlations among social-demographic characteristics, depression, anxiety, symptom clusters and QOL.

Variables	Age	Number of relapses	Duration of disease	QOL	Somatosensory symptom	Motor symptom	visual-memory symptom	bladder-rectal symptom	Sleep symptom	Anxiety	Depression	ADL
Age	1											
Number of relapses	0.167*	1										
Duration of disease	0.230**	0.571**	1									
QOL	−0.191*	−0.121	−0.007	1								
Somatosensory symptom	0.369**	0.217*	0.109	−0.276**	1							
Motor symptom	0.325**	0.151	0.049	−0.296**	0.643**	1						
Visual-memory symptom	0.196*	−0.030	0.035	−0.239**	0.405**	0.519**	1					
Bladder-rectal symptom	0.370**	0.272**	0.170*	−0.265**	0.461**	0.594**	0.391**	1				
Sleep symptom	0.278**	0.100	0.142	−0.215*	0.287**	0.361**	0.425**	0.330**	1			
Anxiety	−0.016	0.155	0.055	−0.222**	0.297**	0.361**	0.443**	0.204*	0.353**	1		
Depression	−0.030	0.179*	0.100	−0.212*	0.314**	0.352**	0.443**	0.258**	0.329**	0.816**	1	
ADL	−0.256**	0.003	0.114	0.311**	−0.244**	−0.364**	−0.235**	−0.319**	−0.131	−0.201*	−0.177*	1

*p < 0 0.05; **p < 0 0.01 (Bonferroni-corrected).

### 3.5 Model test

**Figure 1:**
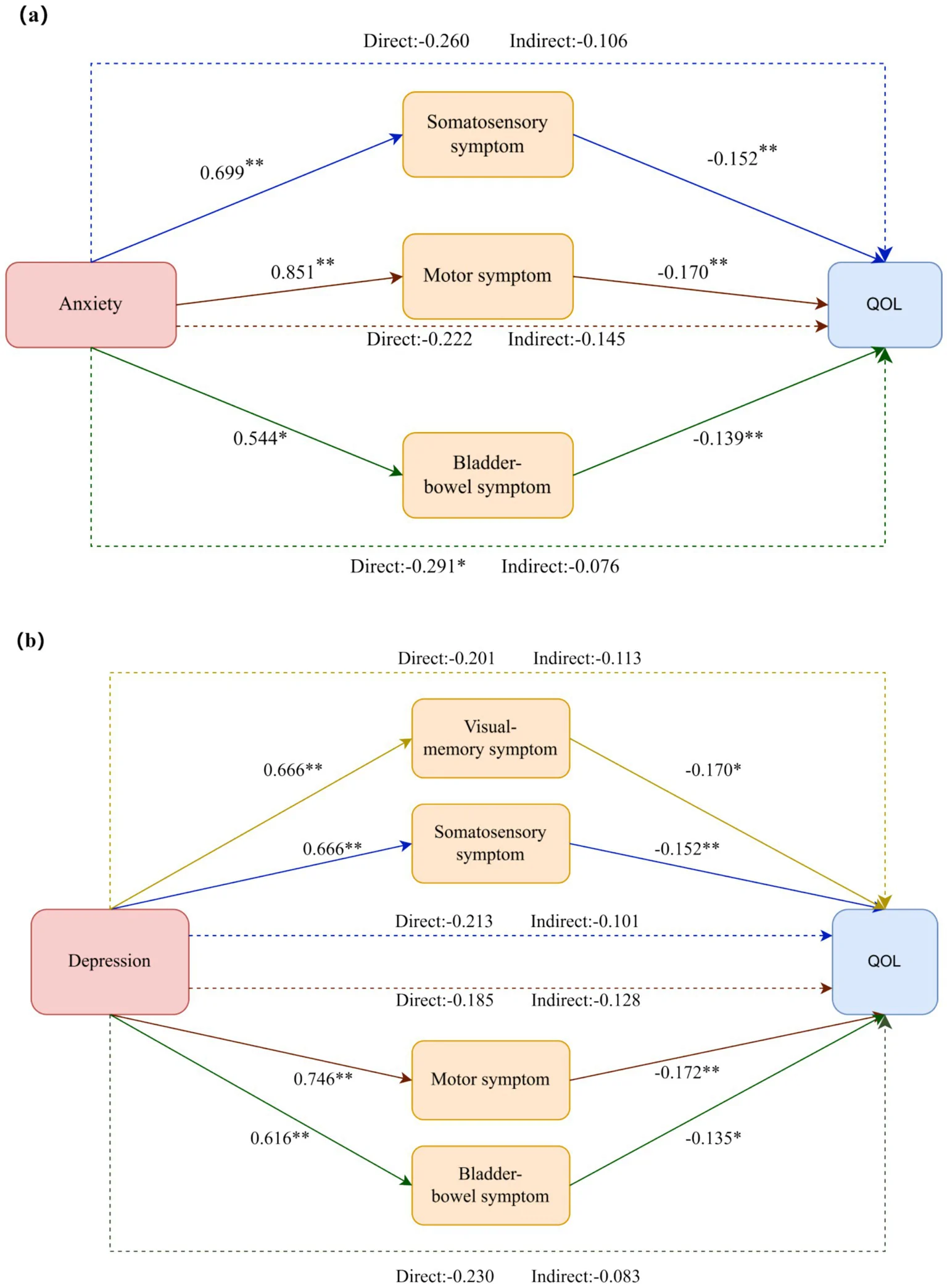
**(a)** Anxiety model; **(b)** Depression model. Mediating effects of symptom clusters between anxiety, depression, and QOL.

Covariates including educational level and primary caregivers were rigorously controlled. Mediation analyses revealed distinct patterns for anxiety and depression pathways (Table 4). For anxiety, somatosensory and motor symptom clusters fully mediated its association with QOL, with indirect effects of *β* = −0.106 (95% *CI*: −0.221, −0.020) and β = −0.145 (95%*CI*: −0.272, −0.034), respectively, and nonsignificant direct effects (somatosensory: *β* = −0.260, *p >* 0.05; motor: *β* = −0.222, *p >* 0.05). In contrast, bladder-rectal clusters partially mediated anxiety’s impact [*β* = −0.076, 95% *CI*: (−0.173, −0.007)], as the direct effect remained significant (*β* = −0.291, *p* = 0.018) (see Figure 1a).

**Table 4:** Mediating effects of symptom clusters between anxiety, depression and QOL.

Indirect effect^a^	Effect of X on M	Effect of M on Y	Direct effect	Indirect effect	Total effect	Effect size	Bootstrapping (BC 95%CI)	
							Lower	Upper
Anxiety→Somatosensory symptom→QOL	0.699**	−0.152**	−0.260	−0.106	−0.366	1.000	−0.221	−0.020
Anxiety→Motor symptom→QOL	0.851**	−0.170**	−0.222	−0.145	−0.366	1.000	−0.272	−0.034
Anxiety→Visual-memory symptom→QOL	0.750**	−0.166*	−0.242	−0.125	−0.366	0.000	−0.168	0.001
Anxiety→bladder-rectal symptom→QOL	0.544*	−0.139**	−0.291*	−0.076	−0.366	0.208	−0.173	−0.007
Anxiety→Sleep symptom→QOL	0.702**	−0.127	−0.278	−0.089	−0.366	0.000	−0.122	−0.005
Depression→Somatosensory symptom→QOL	0.666**	−0.152**	−0.213	−0.101	−0.314	1.000	−0.146	−0.015
Depression→Motor symptom→QOL	0.746**	−0.172**	−0.185	−0.128	−0.314	1.000	−0.164	−0.022
Depression→Visual-memory symptom→QOL	0.666**	−0.170*	−0.201	−0.113	−0.314	1.000	−0.171	−0.004
Depression→bladder-rectal symptom→QOL	0.616**	−0.135*	−0.230	−0.083	−0.314	1.000	−0.129	−0.001
Depression→Sleep symptom→QOL	0.576**	−0.132	−0.238	−0.076	−0.314	0.000	−0.123	−0.005

M, somatosensory/motor/visual-memory/bladder-rectal/sleep symptom; X, anxiety/depression; Y, QOL.

^a^Adjusted for educational level and primary caregivers.

*p < 0.05; **p < 0.01 (Bonferroni-corrected).

Depression exhibited full mediation through four symptom clusters: somatosensory [*β* = −0.101, 95% *CI*: (−0.146, −0.015)], motor [*β* = −0.128, 95% *CI*: (−0.164, −0.022)], visual-memory [*β* = −0.113, 95% *CI*: (−0.171, −0.004)], and bladder-rectal [*β* = −0.083, 95% *CI*: (−0.129, −0.001)], with all direct effects nonsignificant (*p* > 0.05). No significant mediation emerged for depression via sleep clusters [indirect *β* = −0.076, 95% *CI*: (−0.123, 0.005); direct *β* = −0.238, *p >* 0.05)] (see Figure 1b). Sensitivity analyses using 5,000 bootstrap resamples confirmed the robustness of these findings.

## 4 Discussion

In this study, the mean QOL score among NMOSD patients was 97.76 ± 8.24, which is higher than the findings reported by Wang et al. (39) This discrepancy may be attributed to the online research design employed in their study, which could have introduced selection bias toward younger non-inpatient or outpatient individuals with milder symptoms. Additionally, our participants experienced higher levels of anxiety, depression, and symptom distress. Variations in QOL were also observed among NMOSD patients with different educational backgrounds and primary caregiver statuses.

This study examined the association between anxiety/depression and QOL in NMOSD patients, with a specific focus on the mediating effects of symptom clusters. The results demonstrated significant negative correlations between anxiety/depression and QOL, mediated through distinct symptom clusters. Anxiety was associated with QOL, with somatosensory and motor symptom clusters showing full mediation, and bladder-rectal symptoms showing partial mediation. Similarly, depression was indirectly associated with QOL through somatosensory, motor, visual-memory, and bladder-rectal symptom clusters. Sleep-related symptoms did not show significant mediating effects in either pathway. These findings suggest that the associations between anxiety/depression and QOL may operate through indirect pathways involving symptom clusters. This pattern reflects a complex interplay between psychological factors and physical symptoms and underscores the importance of addressing both domains in clinical management.

The present study revealed a significant association between educational level and QOL in NMOSD patients. Analysis showed that patients with intermediate education (secondary school) and higher education (university or above) had significantly higher composite QOL scores compared to other groups, with the highest scores observed in the junior high school subgroup. The relatively stable QOL observed in the higher education group may be related to their greater ability to access health information, utilize healthcare resources, and adopt cognitive coping strategies for chronic disease management (40). Notably, the higher QOL observed in the junior high school subgroup may suggest a more complex pattern: although individuals with higher education tend to have greater medical literacy, increased disease-related awareness may also be associated with greater psychological burden (41). These findings suggest that educational attainment may be associated with QOL in a non-linear manner, potentially involving multiple interacting factors such as social support, psychological stress, and perceptions of disease burden. Future studies with larger samples are needed to further explore these associations and underlying mechanisms.

The present findings demonstrate a significant association between primary caregivers and QOL in NMOSD patients. Comparative analysis revealed comparable QOL scores between self-managed care and kin-supported care groups, both exceeding those observed in non-specialized caregiver cohorts. The lower QOL observed in the latter group may be related to relatively limited disease management knowledge and psychosocial support capacity within informal caregiving contexts. Notably, while kin-supported care provides essential functional assistance, it may inadvertently contribute to autonomy restriction through overprotective behaviors (42). Conversely, self-managing patients may benefit from enhanced self-determination capacity, as reported in previous studies (43). Perceived control over disease management can buffer against the psychological impact of functional disability (44). Clinically, excessive substitute care should be avoided to enhance patients’ self-determination and functional autonomy. This preliminary analysis did not account for critical moderating variables including disease activity status and motor impairment severity. Subsequent investigations should incorporate these parameters to disentangle caregiving modality effects from confounding neurological determinants.

The study findings demonstrate that somatosensory symptom clusters (pain, paresthesia, and constipation) mediated entirely the relationship between anxiety and QOL in NMOSD patients. Anxiety likely amplifies somatic symptom burden through heightened noradrenergic activity, which impairs parasympathetic modulation and potentiates pain perception (45–48). This mechanism establishes a self-perpetuating cycle where prolonged pain exacerbates anxiety, collectively aggravating physiological distress and eroding psychological resilience, ultimately culminating in profound QOL deterioration. Motor symptom clusters (motor dysfunction, ataxia, myasthenia, and limb spasticity) similarly exhibited significant mediation effects. Anxiety-induced hypervigilance toward motor limitations likely exacerbates functional decline through activity avoidance and independence loss. These observations are consistent with prior evidence that anxiety is associated with sensorimotor integration and functional performance, which in turn correlates with QOL (49). This suggests anxiety transcends psychological distress by interacting bidirectionally with motor dysfunction to influence functional status. Partial mediation effects were observed for bladder-rectal symptom clusters (dysuria, urinary frequency/urgency/nocturia, retention, and defecatory incontinence). While epidemiological studies corroborate anxiety’s association with lower urinary tract symptoms, the precise physiological pathways remain undefined (50). Heightened awareness associated with anxiety may relate to shorter voiding intervals, which are correlated with limitations in social participation and QOL. Notably, no significant mediation emerged for visual-memory or sleep-related symptom clusters. This null association may reflect either the limited sample size or intrinsic low sensitivity of these clusters to anxiety pathways. Future multicenter longitudinal studies with expanded cohorts are needed to validate these relationships. In conclusion, anxiety is associated with NMOSD-related QOL, in relation to distinct symptom networks. Clinical protocols must prioritize systematic anxiety screening and implement cluster-specific interventions: cognitive-behavioral strategies coupled with physiotherapy for somatosensory/motor clusters, and structured bladder-rectal retraining for excretory dysfunction. A tiered intervention framework addressing both psychological and somatic dimensions may optimize functional outcomes in this population.

This study revealed the pattern of associations between depression and QOL through path analysis. The results showed that depression had a fully mediated effect on QOL through somatosensory, motor, visual-memory, and bladder-rectal symptom clusters. The pathways of action are characterized by the following features: previous studies have shown that depressed patients tend to exhibit heightened sensitivity to pain, and that this heightened perception exacerbates the patients’ somatic discomfort, which aligns with the findings of the present study (47). Depression and pain potentially share associations through common neuroinflammatory mechanisms (such as disordered excitatory glutamate metabolism or inhibitory *γ*-aminobutyric acid and glycine metabolism) and common neuroanatomical pathways and structures (such as the hippocampus and nucleus accumbens) (51, 52). Unlike anxiety, depression is more often characterized by persistent low mood and pessimism about the future, an emotional state that may make patients less tolerant of somatic symptoms, thus amplifying their negative impact on QOL.

Compared to anxiety, depressed patients may exhibit a more pronounced lack of motivation and loss of interest, which is associated with reduced participation in rehabilitation and social activities, and is further linked to motor symptom clusters and QOL. Previous studies have found that depression is associated with gastrointestinal dysfunction, overactive bladder and lower urinary tract symptoms, which is consistent with the results of the present study suggesting an association between depression and bladder-rectal symptom clusters (50, 53–55). The results of the present study also showed that depression was indirectly associated with QOL through visual-memory symptom clusters (visual disturbances, diplopia, memory loss, and dizziness), which may relate to alterations in neurotransmitter levels (e.g., dopamine and norepinephrine) associated with depression, which are involved in the processing of visual information, and to the possibility that patients with depression may exhibit delayed perception of visual information in relation to distraction or low mood. Additionally, previous studies (56) have shown that depression is associated with cognitive impairment; visual impairment is closely related to patients’ daily life, while cognitive impairment is associated with decision-making ability and socialization. Together, these symptoms are associated with reduced social participation and independence, and are further linked to lower QOL, consistent with previous studies showing an association between visual impairment and reduced QOL (57).

Anxiety and depression are significantly associated with QOL in NMOSD patients through multiple symptom clusters, suggesting that the synergistic effects of psychosocial and somatic factors need to be integrated into clinical management to improve patients’ somatic symptoms and QOL. Future studies are needed to further validate these findings and explore appropriate intervention strategies to better address the clinical needs of NMOSD patients. Additionally, if more cases can be collected in future research, parallel mediation analysis could be employed to compare the effects of multiple mediating pathways and identify key pathways with stronger effects.

## 5 Limitations

Although the present study provides valuable insights into the mediating effects of anxiety, depression, and symptom clusters in the QOL of patients with NMOSD, several limitations remain. First, the study utilized a cross-sectional design, which did not allow for the determination of causality. Future studies could further validate the mediating role of symptom clusters between anxiety and depression and QOL through a longitudinal design. Second, the study sample was mainly from China, and there may be geographical and cultural differences that limit the extrapolation of the results. Third, although optic nerve disorders are rare diseases, the sample size of 140 cases may affect the reliability of the results, and future studies should expand the sample size to verify the generalizability of the results. Fourth, there is no symptom scale in the literature for patients with NMODS; therefore, a self-administered questionnaire was used in this study. Although the NMOSD Symptom Scale showed good reliability and validity in the current study, more studies are needed to prove its validity.

## 6 Conclusion

This study revealed that anxiety and depression are significantly associated with QOL in patients with NMOSD through distinct symptom clusters. Anxiety was indirectly associated with QOL primarily through somatosensory and motor symptom clusters, with partial mediation via bladder-rectal symptoms. Depression was indirectly associated with QOL through somatosensory, motor, visual-memory, and bladder-rectal symptom clusters. These findings highlight the mediating role of symptom clusters in the relationship between psychological distress and QOL, suggesting a shift from symptom-specific management approaches to cluster-based intervention models, and the importance of considering integrated psychological and somatic management strategies in clinical practice. Future research is needed to validate these findings in larger, more diverse cohorts and to explore targeted interventions aimed at improving outcomes for NMOSD patients.

## Data Availability

The raw data supporting the conclusions of this article will be made available by the authors, without undue reservation.

## Footnotes

1
https://weixin.qq.com/
