# Supplementary material for: Symptom clusters mediate anxiety/depression effects on quality of life in neuromyelitis optica spectrum disorders: a cross-sectional mediation analysis
Source: Front Neurol. 2026 Jun 18;17:1753642. doi: 10.3389/fneur.2026.1753642 (PMC13325464; doi:10.3389/fneur.2026.1753642)
Supplement: Supplementary file 1 [file Table_1.DOCX]

Patients access for eligibility

(n=206)

Declined (n=55)

Nurses after eligibility examination

(n=151)

Nurses excluded (n=11):

<18 years old (n=5)

Pregnant or breastfeeding women (n=4)

malignant tumors (n=1)

diagnosed with dementia (n=1)

Valid sample

(n=140)

Flowchart of participants selection process
